# Cardiovascular–Kidney–Metabolic Syndrome and Its Hepatic Dimension: A Narrative Review

**DOI:** 10.3390/medicina62091742

**Published:** 2026-09-10

**Authors:** Vasilica Enache, Dan-Cristian Popescu, Bogdan Marcu, Mara Diaconu, Alexandru-Cristian Nechita

**Affiliations:** 1 Department of Cardiology, Clinical Emergency Hospital Sfântul Pantelimon, 021659 Bucharest, Romania; vasi.enache@eucardios.ro (V.E.); popescu_dan_95@yahoo.com (D.-C.P.); bogdan.marcu18@gmail.com (B.M.); cardionechita@yahoo.com (A.-C.N.); 2 Faculty of Medicine, Carol Davila University of Medicine and Pharmacy, 020021 Bucharest, Romania; 3 Faculty of Medicine, Transilvania University, 500036 Brasov, Romania

**Keywords:** metabolic syndrome, cardiovascular-kidney-metabolic syndrome, metabolic dysfunction-associated steatotic liver disease, insulin resistance, miRNA, integrated therapy

## Abstract

Metabolic syndrome came to the attention of the scientific community several decades ago, and its definition has undergone multiple changes over time. It is currently defined by the coexistence of hypertension, central obesity, dyslipidemia, and impaired glucose metabolism, with insulin resistance, chronic inflammation, and oxidative stress representing important underlying pathophysiological mechanisms. The latter two processes are major promoters of the onset and progression of atherosclerosis. In parallel, many individuals develop metabolic dysfunction-associated steatotic liver disease (MASLD), formerly called non-alcoholic fatty liver disease (NAFLD). Growing evidence indicates that MASLD may interact bidirectionally with cardiovascular, renal, and metabolic dysfunction and may contribute to cardiometabolic risk. These observations have prompted proposals for a broader cardio–reno–hepato–metabolic axis. However, MASLD is not currently included in the established cardiovascular-kidney-metabolic (CKM) definition or staging system, and its incorporation remains an evolving conceptual extension. Prediabetes is a reversible condition characterized by abnormal glucose levels that do not meet the diagnostic criteria for diabetes. The American Diabetes Association defines prediabetes as glycated hemoglobin (HbA1c) = 5.7–6.4%, fasting plasma glucose = 100–125 mg/dL, or two-hour plasma glucose during an oral glucose tolerance test = 140–199 mg/dL. This comprehensive review examines traditional and genetic risk determinants, shared pathophysiological mechanisms, diagnostic strategies, clinical manifestations, emerging phenotypes, circulating microRNAs as non-invasive biomarkers, complications, and the principal therapeutic strategies, including diet, exercise, and pharmacotherapy. Particular attention is given to the recent approvals of resmetirom and semaglutide for metabolic dysfunction-associated steatohepatitis and to the expanding roles of sodium-glucose cotransporter 2 (SGLT2) inhibitors, glucagon-like peptide-1 (GLP-1) receptor agonists and non-steroidal mineralocorticoid receptor antagonists. Cardiovascular, renal, metabolic, and hepatic disorders are frequently present in the same patient. Other individuals may develop this high-risk cluster over time. The aim of this article is to define the interplay between different metabolic conditions and to outline the best approach to diagnosis, monitoring, and effective integrated therapy.

## 1. Introduction: Definitions, Staging and Nomenclature

Cardiovascular–kidney–metabolic (CKM) syndrome is defined as a systemic disorder arising from the pathophysiological interactions among metabolic risk factors, including obesity and type 2 diabetes mellitus (T2DM), chronic kidney disease (CKD) and cardiovascular disease (CVD), leading to substantial morbidity and premature mortality, with a disproportionate burden of cardiovascular events [1,2]. The CKM framework moves beyond the management of individual risk factors towards an integrated staging system that reflects the continuum of risk and identifies opportunities for prevention [2]. This framework was formalized in the 2026 American Heart Association/American College of Cardiology/American Diabetes Association/American Society of Nephrology (AHA/ACC/ADA/ASN) clinical practice guideline, which established CKM syndrome as a living, working clinical construct comprising Stages from 0 to 4 [1].

The National Cholesterol Education Program Adult Treatment Panel III (NCEP ATP III) criteria remain among the most widely used definitions of metabolic syndrome in clinical practice and are presented in Table 1 [3,4].

Alternative definitions were proposed by the International Diabetes Federation (IDF), the World Health Organization (WHO), and the European Group for the Study of Insulin Resistance (EGIR) (Table 2) [5,6,7,8,9]. The harmonized definition removed the requirement for abdominal obesity as a mandatory component and refined the NCEP ATP III criteria by introducing population- and ethnicity-specific waist circumference thresholds [10].

The 2026 AHA/ACC/ADA/ASN guideline uses a five-stage CKM framework spanning the continuum from risk factor prevention to established CVD. Stage 0 denotes the absence of CKM risk factors. Stage 1 includes excess or dysfunctional adiposity, insulin resistance, or prediabetes. Stage 2 comprises established metabolic risk factors, such as T2DM, hypertension, hypertriglyceridemia, or metabolic syndrome, as well as moderate-to-high-risk CKD. Stage 3 represents subclinical CVD in the CKM context or very-high-risk CKD. Stage 4 represents established clinical CVD in the CKM context and is subdivided according to the presence or absence of kidney failure [1,2]. CKM assessment across the life course is intended to facilitate early prevention and the tailoring of therapy to absolute risk [1]. Importantly, hepatic disease is not included as a formal component of the current AHA CKM definition or staging system.

The proposed incorporation of the hepatic dimension into the CKM framework represents an emerging conceptual development. Metabolic dysfunction-associated steatotic liver disease (MASLD) was introduced in 2023 to replace non-alcoholic fatty liver disease (NAFLD), removing potentially stigmatizing terminology and explicitly linking the disease to its metabolic basis. Its inflammatory form is termed metabolic dysfunction-associated steatohepatitis (MASH) [11]. The 2023 multisociety Delphi consensus replaced NAFLD and non-alcoholic steatohepatitis (NASH) with MASLD and MASH, respectively. MASLD is defined by hepatic steatosis together with at least one of five cardiometabolic criteria and alcohol consumption below the metabolic dysfunction- and alcohol-related steatotic liver disease (MetALD) range (Table 3) [11,12]. Depending on the patient’s metabolic profile, alcohol intake, and underlying etiology, other categories within the steatotic liver disease nomenclature include MetALD and cryptogenic steatotic liver disease (SLD) [11].

Because hepatic steatosis may arise early in the cardiometabolic continuum and may itself exacerbate insulin resistance and vascular risk, several authors consider the absence of hepatic variables from the original AHA staging system to be a potential limitation. Proposals such as the liver–metabolic–vascular construct seek to position the liver more explicitly within this physiopathological cascade [15]. Accordingly, recent literature has proposed a broader “cardio–reno–hepato–metabolic axis” to emphasize these biological and clinical interactions [16]. Throughout this review, this term is used as a conceptual framework rather than as a formally recognized syndrome or an alternative to the established AHA CKM classification.

## 2. Materials and Methods

This narrative review was based on a structured search of PubMed, Scopus, and Web of Science, covering studies up until July 2026. Search terms included combinations of “cardiovascular–kidney–metabolic syndrome”, “CKM syndrome”, “metabolic syndrome”, “MASLD”, “MASH”, “NAFLD”, “chronic kidney disease”, and “cardiovascular disease”, together with terms related to epidemiology, pathophysiology, diagnosis, biomarkers, phenotypes, and therapy. Recent evidence published between 2020 and 2026 was prioritized, while earlier landmark studies were retained for historical definitions, diagnostic methods, and foundational pathophysiological concepts. Eligible publications included clinical guidelines, consensus statements, systematic reviews, meta-analyses, clinical trials, observational studies, and relevant mechanistic studies. Case reports, conference abstracts, non-peer-reviewed publications, and articles outside the scope of the review were excluded. Clinical trial registry entries were used only to describe registered studies, not as evidence of treatment efficacy. Titles and abstracts were screened for relevance, followed by full-text assessment. Because of the narrative design and heterogeneity of the included evidence, findings were synthesized qualitatively without meta-analysis or formal risk-of-bias assessment.

## 3. Epidemiology

### 3.1. Epidemiology of CKM

Population-level estimates of CKM syndrome are based on the distribution of AHA-defined CKM stages and should be distinguished from the prevalence estimates of individual organ diseases. A 2024 analysis of the U.S. National Health and Nutrition Examination Survey (NHANES, 2011–2020) reported that approximately 90% of U.S. adults met criteria for at least Stage 1 [17]. Because Stage 1 includes excess or dysfunctional adiposity, this estimate reflects the prevalence of individuals across the CKM risk continuum and should not be interpreted as the prevalence of established cardiovascular, kidney, or hepatic disease. A 2026 systematic review and meta-analysis pooling more than ten million participants confirmed that mortality risk rises monotonically with advancing CKM stage, providing the prognostic validation the staging system required [18].

### 3.2. Epidemiology of MASLD

In contrast to CKM stage estimates, MASLD prevalence is determined by the presence of hepatic steatosis together with at least one cardiometabolic risk criterion. Global Burden of Disease (GBD) analyses estimate roughly 1.27 billion prevalent cases in 2021, with an age-standardized prevalence rising by about 24% since 1990 [19,20]; forecasts project the burden to reach 1.8 billion cases by mid-century if current trends persist [21]. High fasting plasma glucose and smoking are identified as the leading attributable risk factors [19]. Overall, MASLD affects approximately one-third of the global adult population [22,23]. Although CKM syndrome and MASLD share several metabolic determinants and frequently coexist, their epidemiological estimates are not interchangeable because they are based on different definitions, populations, and outcome measures.

#### 3.2.1. United States of America

In the United States, MASLD prevalence shows a marked ethnic gradient. Hispanic and Mexican–American populations show the highest hepatic steatosis prevalence—NHANES vibration-controlled transient elastography data placed Mexican–American steatosis near 46.7%, the highest of any group assessed—reflecting the interaction of genetic susceptibility and metabolic load [24].

#### 3.2.2. Latin America

Latin America carries one of the heaviest relative burdens. Regional estimates place MASLD prevalence as high as ~44%, driven by high rates of obesity and T2DM, energy-dense diets, low physical activity, and genetic predisposition (notably *PNPLA3* risk allele frequency in admixed populations) [24]. GBD data assign Andean and Central Latin America the highest MASLD-attributable death and disability-adjusted life year (DALY) rates globally, with Mexico reporting among the highest national DALY rates; liver-related mortality in the region exceeds that of much of Asia and Africa [19,24]. Underdiagnosis is severe because biopsy and magnetic resonance imaging are frequently unavailable in routine care [24].

#### 3.2.3. Europe

Western Europe shows a high age-standardized MASLD prevalence with continued growth since 1990 [19]. European observational studies have contributed to characterizing the association between steatotic liver disease and cardiovascular outcomes [23]. Broader evidence also supports an association between MASLD and CKD and the use of non-invasive fibrosis assessment for risk stratification [16]. Notably, resmetirom received U.S. approval in 2024 and conditional marketing authorization in the European Union in August 2025 [25].

#### 3.2.4. Africa

Africa shows comparatively lower measured MASLD prevalence, on the order of ~20% [26]. Paradoxically, North Africa and the Middle East register the highest age-standardized prevalence rates worldwide—Kuwait reported the single highest national rate in GBD analyses—reflecting rapid nutritional transition, high T2DM burden, and obesity [19].

#### 3.2.5. Asia

Asian estimates cluster around 28–32% for MASLD, with substantial heterogeneity [26]. The high-income Asia–Pacific subregion has among the lowest age-standardized prevalence and DALY rates, while East Asia also recorded one of the lowest DALY rates in recent GBD reporting [19]. Analyses of UK and Taiwan Biobank data have also contributed to the genetic and phenotypic clustering work discussed below [27].

## 4. Traditional Risk Factors

The conventional drivers of the syndrome are the components of the metabolic syndrome themselves. Excess and dysfunctional adiposity—particularly visceral and ectopic fat—is a key upstream driver in the CKM staging model and a major driver of hepatic steatosis [2,22]. Insulin resistance and T2DM amplify hepatic de novo lipogenesis, glomerular hyperfiltration, and atherogenesis; the coexistence of MASLD and T2DM produces a cumulative, amplified pathogenic effect on cardiovascular and renal endpoints rather than a simple sum of risks [16]. Atherogenic dyslipidemia (elevated triglycerides, low HDL cholesterol) and hypertension complete the classical cluster [3,4].

Additional traditional contributors documented in the recent literature include high fasting plasma glucose, tobacco use, physical inactivity, energy-dense nutrient-poor dietary patterns, and—increasingly recognized—alcohol intake interacting with metabolic risk in steatotic liver disease [12,19]. Aldosterone excess and a high atherogenic index of plasma have been proposed as additional, measurable risk markers for MASLD [22].

## 5. Genetic Determinant Factors

Heritable susceptibility is well established, especially for the hepatic component [28]. The patatin-like phospholipase domain-containing 3 gene variant *PNPLA3* rs738409 (I148M) is a major common genetic risk factor for steatotic liver disease, associated with greater hepatic fat content, higher transaminases, and progression to significant fibrosis, cirrhosis, and hepatocellular carcinoma [29,30]. The *TM6SF2* rs58542926 (E167K) variant, which impairs very-low-density lipoprotein assembly and secretion, independently increases hepatic triglyceride retention; additional steatosis/fibrosis loci include *MBOAT7*, *GCKR*, *HSD17B13*, and *SIRT5* [28,31]. Genetic susceptibility has also been demonstrated in pediatric populations and Asian cohorts, including Chinese and Thai populations [29,31,32].

Two recent conceptual advances are important. First, gene–environment interaction: *PNPLA3* and *TM6SF2* are not fixed, independent determinants but amplifiers—they exacerbate the harmful effects of obesity, insulin resistance, and alcohol, while modifiable factors can attenuate genetic risk, supporting individualized counseling and risk stratification [28,33]. Second, partitioned polygenic risk scores have shown that MASLD is genetically heterogeneous: some leading hepatic fat alleles do not raise cardiovascular risk, distinguishing a “steatosis-predominant” genetic architecture from a “cardiometabolic” one and arguing against a uniform causal link between liver fat and vascular disease [28,34].

*TRIB1* and *SLC39A8* illustrate the potentially divergent hepatic and cardiovascular effects of genetic variation. *TRIB1* participates in lipid metabolism and triglyceride regulation, providing a plausible link to atherosclerotic disease. *SLC39A8* encodes a metal transporter implicated in inflammatory signaling and oxidative stress pathways [28,35]. In an analysis of 5244 elderly Prospective Study of Pravastatin in the Elderly at Risk (PROSPER) participants, *TRIB1* rs2954021 was associated with fatal or non-fatal stroke, while *SLC39A8* rs13107325 was associated with fatal or non-fatal myocardial infarction [35].

In the same study, homozygous variant genotypes at *SLC39A8* rs13107325, *TRIB1* rs2954021, and *ANPEP* rs7168849 were associated with lower Fibrosis-4 (FIB-4) scores, whereas *PNPLA3* rs738409 and *SAMM50* rs3761472 were associated with higher scores. However, the absolute FIB-4 values generally remained within low-risk ranges, and lower scores should not be interpreted as established protection against fibrosis or cardiovascular disease [35]. These observational findings suggest variant-specific systemic effects but do not establish causality or validated cardiovascular mortality prediction in patients with confirmed MASLD/MASH. Their applicability requires confirmation in well-characterized hepatic cohorts, with attention to genotype, ancestry, and the distinction between surrogate fibrosis markers and clinical outcomes.

## 6. Pathophysiology

The hepatic component of this continuum can be understood through the “multiple parallel hit” hypothesis. Rather than requiring a sequential progression from steatosis to a single subsequent inflammatory insult, this model proposes that adipose tissue dysfunction, insulin resistance, gut-derived signals, lipotoxicity, organelle stress, and genetic susceptibility interact to promote hepatic inflammation and fibrosis [36]. These processes may develop concurrently and reinforce one another, with their relative contributions differing between individuals.

Recent mechanistic work, synthesized in a 2025 review, extends this framework by linking gut-derived substrates, including acetate and ethanol, and adipose-derived fatty acids to hepatic de novo lipogenesis and altered mitochondrial metabolism. Increased oxidative activity may initially accommodate substrate overload, whereas impaired mitochondrial function with disease progression can amplify hepatocellular injury [37]. Complementary genetic evidence identifying distinct hepatic and cardiometabolic forms of MASLD further supports heterogeneous disease pathways rather than a uniform sequence of injury [34]. Together, these findings refine the multiple parallel hit model, although individual mechanisms have varying levels of experimental and clinical support.

The pathophysiological continuum linking metabolic, cardiovascular, renal, and hepatic disorders is better understood as a network of self-reinforcing inter-organ interactions than as a series of isolated pathways. Dysfunctional visceral adipose tissue increases free-fatty acid flux and the release of pro-inflammatory mediators [38,39,40]. Reduced adiponectin further weakens insulin sensitivity and anti-inflammatory signaling [41,42]. These changes aggravate systemic insulin resistance and promote hepatic de novo lipogenesis, oxidative stress, and lipotoxicity [40,43]. The steatotic and inflamed liver may subsequently amplify systemic dysfunction through the release of inflammatory and profibrotic mediators [43]. Atherogenic lipoproteins promote endothelial injury and atherosclerosis, while higher circulating advanced glycation end-product levels have been associated with more advanced CKM stages and adverse stage transitions [44,45,46].

At the cardiovascular level, circulating metabolic and inflammatory signals promote endothelial dysfunction and atherosclerotic plaque progression [46,47,48]. Oxidative stress contributes to vascular injury and plaque instability, while chronic inflammation further amplifies cardiovascular damage [49,50]. Together, these mechanisms link metabolic and hepatic dysfunction with atherosclerotic cardiovascular disease and heart failure and may reinforce renal and hepatic injury as part of a bidirectional multiorgan loop [16,23,51].

The liver–kidney interaction represents a particularly relevant component of this multisystem continuum. Recent evidence indicates that MASLD is associated with both incident CKD and CKD progression through mechanisms extending beyond their shared cardiometabolic risk factors [52]. Hepatic insulin resistance and lipid accumulation promote the release of pro-inflammatory cytokines, reactive oxygen species, and pro-fibrotic mediators, which may contribute to renal endothelial and tubular injury. Renin–angiotensin system activation further links the two organs: angiotensin II promotes hepatic lipogenesis, inflammation, and fibrosis, while renal activation contributes to efferent arteriolar vasoconstriction, abnormal lipid accumulation, oxidative stress, glomerulosclerosis, and progressive renal dysfunction [52]. These interconnected pathways support the clinical evaluation of hepatic disease in patients at risk of CKD, while not implying that MASLD is formally incorporated into current CKM staging [1,2,52].

Nevertheless, the association of hepatic steatosis with cardiovascular and renal disease should not be interpreted as uniformly causal. Genetic studies indicate that MASLD comprises biologically distinct phenotypes and that some variants associated with increased hepatic fat do not confer a parallel increase in cardiometabolic risk [28,34]. Systemic risk may therefore depend more strongly on the underlying metabolic, inflammatory, and fibrotic context than on steatosis alone [28,34].

## 7. Diagnosis

Diagnosis requires staged, multi-organ assessment rather than a single test. For the metabolic and cardiovascular axes, the AHA framework recommends systematic CKM staging that incorporates adiposity measures, glycemia, lipid profile, and blood pressure, together with the assessment of albuminuria using the urine albumin-to-creatinine ratio and the estimation of glomerular filtration rate, because kidney injury is frequently silent and under-screened in primary care [1,2]. Composite indices such as the triglyceride–glucose (TyG) index, the atherogenic index of plasma, the cardiometabolic index, and cardiac risk ratios have been investigated as low-cost surrogate markers for cardiometabolic risk stratification [53].

For the hepatic axis, liver biopsy remains the histological reference standard but is invasive and impractical for population-level assessment [12,54]. Consequently, non-invasive tests predominate (Table 4) [12,54,55]. Serum-based scores, particularly the FIB-4 index, are used for first-line risk stratification, followed by vibration-controlled transient elastography (VCTE, FibroScan, including the controlled attenuation parameter) and, where available, magnetic resonance imaging–proton density fat fraction or magnetic resonance elastography [12,54,55]. FIB-4 is recommended as a first-line assessment in primary care and diabetology because of its simplicity and low cost [12,54]. *PNPLA3* genotyping has been investigated as an adjunct to FIB-4 and clinical risk factors for prediction of future severe liver disease, although its incremental clinical value remains incompletely established [30].

## 8. Clinical Picture

The syndrome is typically asymptomatic during its early and intermediate stages, which is precisely why active screening and staging are emphasized. Abdominal obesity can be assessed by measuring waist circumference, while bioelectrical impedance can provide additional estimates of body composition and visceral adiposity [1]. As the syndrome advances, clinical manifestations reflect the predominantly affected organs: exertional dyspnea and signs of heart failure, frequently heart failure with preserved ejection fraction (HFpEF), angina or previous myocardial infarction, or atrial fibrillation on the cardiovascular side [1,23]; increasing albuminuria, declining estimated glomerular filtration rate (eGFR) and ultimately kidney failure on the renal side [1,16]; and progressive fibrosis and eventually the stigmata of cirrhosis or hepatocellular carcinoma on the hepatic side [12]. Because subclinical disease frequently coexists in several organs, the clinical picture is best understood as a continuum of cumulative, multi-organ risk rather than as a discrete presentation [1,2].

## 9. Phenotypes

Patients meeting identical diagnostic thresholds can differ markedly in biology and prognosis, motivating phenotypic stratification. A clinically intuitive division contrasts the metabolically unhealthy obese (MUHO) with the metabolically unhealthy normal-weight (“lean”) phenotype; recent work shows that cardiovascular risk indices, including TyG-derived indices and the atherogenic index of plasma, are significantly higher in MUHO than in metabolically unhealthy normal-weight (MUHNW) individuals [53,60].

Unsupervised machine learning approaches have refined this further. Clustering of biobank data (UK and Taiwan) identified reproducible metabolic syndrome endotypes—for example, non-descriptive, hypertensive, obese, lipodystrophy-like, and hyperglycemic clusters—each with distinct trait profiles, genetic associations, and outcome risks [27]. Korean Korea National Health and Nutrition Examination Survey (KNHANES) “metabotype” analyses derived low-, intermediate-, and high-risk clusters from routine biomarkers (body mass index [BMI], uric acid, fasting glucose, high-density lipoprotein [HDL], and non-HDL cholesterol) [61]. Together, these data support phenotype-based risk stratification and the development of more personalized prevention strategies [27,53,61].

## 10. MicroRNAs as Investigational Biomarkers

Circulating microRNAs (miRNAs) are being investigated as non-invasive biomarkers across hepatic, renal, and cardiometabolic disorders [62,63,64]. In MASLD, liver-enriched miR-122 and several other miRNAs have been associated with steatosis and fibrosis severity, while extracellular vesicle miRNA profiles are being explored across different disease stages [62,65,66]. In early diabetic kidney disease, pooled evidence suggests moderate diagnostic performance for circulating miRNAs, and cardiometabolic miRNA signatures have also been proposed for early risk stratification [63,64]. However, the available evidence is limited by small and heterogeneous cohorts, differences in sample type and analytical methods, inconsistent normalization procedures and diagnostic thresholds, and limited prospective or external validation [62,63,64]. No miRNA-based assay is currently established for routine CKM or MASLD assessment. Therefore, their clinical role remains investigational and requires validation in large, standardized prospective cohorts [62,63,64].

## 11. Clinical Complications, Associated Conditions and Metabolic Risk States

To avoid conflating conceptually distinct clinical entities, this section differentiates end-organ complications from associated conditions and clinical risk markers, as well as from the intermediate metabolic risk states and progression to T2DM.

### 11.1. End-Organ Complications

#### 11.1.1. Cardiovascular Complications

Cardiovascular complications constitute a major source of morbidity and mortality associated with metabolic syndrome and advanced CKM stages. These include myocardial infarction, stroke, and heart failure, particularly HFpEF, and atrial fibrillation. MASLD/MASH has also been associated with increased cardiovascular risk [16,23]. Pooled data confirm that the syndrome roughly doubles the risk of major cardiovascular events, with a systematic review and meta-analysis of 87 prospective studies reporting an approximately two-fold increase in cardiovascular disease and cardiovascular mortality and a 1.5-fold increase in all-cause mortality; the syndrome was associated with an increased risk of myocardial infarction (risk ratio [RR] 1.99) and stroke (RR 2.27), and importantly this elevated risk persisted even in patients without diabetes [67]. The atherothrombotic burden is concentrated in the ischemic endpoints: among individuals with excess body weight, a more recent meta-analysis of nine cohorts found that metabolic syndrome was significantly associated with myocardial infarction (hazard ratio [HR] 1.68) [68].

Beyond ischemic disease, metabolic syndrome is strongly associated with both HFpEF and atrial fibrillation. HFpEF in particular is now conceptualized less as a hemodynamic disorder than as a systemic metabolic–inflammatory disease: more than 80% of HFpEF patients are overweight or obese, and the clustering of visceral adiposity, insulin resistance, and hypertension promotes left ventricular hypertrophy and diastolic dysfunction [69]. The arrhythmic risk is comparably robust: a meta-analysis of prospective cohorts found that metabolic syndrome was associated with an increased risk of atrial fibrillation (HR 1.57), with several individual components also associated with increased risk [70].

Metabolic syndrome is associated with an increased probability of left ventricular hypertrophy, even after adjustment for hypertension, obesity, and diabetes (odds ratio [OR] 1.55; 95% confidence interval [CI] 1.18–2.04) [71].

Metabolic syndrome is associated with an increased risk of incident heart failure (HR 1.32; 95% CI 1.04–1.68 in non-diabetic patients with established cardiovascular disease), with the risk increasing with the number of components present. Among the components assessed in that cohort, waist circumference was the only independent predictor [72].

#### 11.1.2. Hepatic Complications

Hepatic complications span steatohepatitis, progressive fibrosis, cirrhosis, hepatic decompensation, and hepatocellular carcinoma [16,73]. The coexistence of hepatic and extrahepatic complications supports integrated multidisciplinary management [2,16]. In a large NHANES analysis of the MASLD population, 65% had three or more cardiometabolic disorders. Hypertension, (pre-)diabetes, and hypertriglyceridemia were the strongest predictors of all-cause mortality, whereas increased waist circumference, (pre-)diabetes, and hypertension showed the strongest associations with fibrosis [74]. A distinctive and clinically important feature of MASLD-driven liver disease is that hepatocellular carcinoma (HCC) can arise before cirrhosis is established. Although HCC typically arises in a cirrhotic liver, it occurs in about 20% of cases in the absence of cirrhosis, and among non-cirrhotic causes, NAFLD currently represents the most important emerging driver of HCC in developed countries [73].

#### 11.1.3. Renal Complications

Renal complications progress from albuminuria and declining eGFR to end-stage kidney disease, with MASLD acting as a risk enhancer for incident and progressive CKD [16,52]. Epidemiologically, the presence of the syndrome raises the risk of developing chronic kidney disease roughly 1.4- to 2.5-fold (and as much as 4.4-fold in men) [75]. Crucially, renal involvement often begins before overt disease: disturbances in lipid handling and microalbuminuria can emerge in the earliest stages, marking the kidney as an early rather than late casualty of metabolic dysregulation [75,76]. At the tissue level, the syndrome imprints a characteristic renal phenotype. Obesity-driven hyperfiltration and glomerulomegaly mechanically stretch podocytes beyond their capacity to cover the enlarged glomerular surface, culminating in foot process detachment and focal segmental glomerulosclerosis—the lesion termed obesity-related glomerulopathy—alongside mesangial expansion and podocyte hypertrophy [75]. Ectopic lipid then accumulates within podocytes, mesangial cells, and tubular and interstitial compartments, where it fuels oxidative stress, mitochondrial dysfunction, inflammation, fibrosis, and apoptosis, linking the systemic lipid excess of the syndrome directly to progressive glomerular and tubular injury [76]. Beyond initiating kidney damage, metabolic syndrome also worsens the trajectory of patients who already have CKD, which is where organ-specific outcome data are most informative. In the German Chronic Kidney Disease cohort of 5110 patients with moderately reduced eGFR and/or albuminuria, nearly two-thirds met criteria for the syndrome, and its presence independently predicted higher all-cause mortality (HR ≈ 1.26) and cardiovascular events (HR ≈ 1.48) over 6.5 years, with risk rising in a graded fashion as additional components accumulated. Notably, the individual components were not equivalent: the glucose component carried the greatest weight for both mortality and cardiovascular endpoints, followed by the HDL cholesterol and triglyceride components, whereas elevated triglycerides showed a paradoxical inverse association with all-cause mortality in this CKD population—a reminder that risk relationships established in the general population cannot be extrapolated uncritically to the kidney disease setting [77].

### 11.2. Associated Conditions and Clinical Risk Markers

#### 11.2.1. Erectile Dysfunction

Erectile dysfunction (ED) should be considered an associated vascular risk marker rather than a direct end-organ complication of metabolic syndrome. It frequently precedes clinically overt cardiovascular disease and may indicate underlying systemic endothelial dysfunction [78,79]. Men with metabolic syndrome show markedly higher ED prevalence and substantially poorer endothelial function than matched controls, and ED severity tends to track with the number of metabolic syndrome components a patient carries. Mechanistically, the same disturbances that drive kidney and cardiovascular damage—reduced nitric oxide bioavailability, Rho-associated coiled coil kinase activation, oxidative stress, and low-grade inflammation—act on the small, high-surface-area vessels of the corpus cavernosum, which are especially vulnerable and often show injury before larger vascular beds become symptomatic. This is why ED frequently precedes overt cardiovascular events and can flag heightened cardiometabolic risk years in advance [79]. Two recent studies reinforce ED’s value as a cross-organ marker. In a Taiwanese cohort of 596 men, carriers of the vascular endothelial growth factor (*VEGF*) −2578 A allele had significantly higher odds of both metabolic syndrome and ED, with lower erectile function scores and more metabolic syndrome components as the A-allele count rose—suggesting shared genetic susceptibility involving endothelial VEGF signaling [80]. At the population level, analysis of nearly 100,000 men in the All of Us program directly situated ED within the newly defined cardiovascular–kidney–metabolic (CKM) syndrome: men with ED had substantially higher rates of diabetes, hypertension, chronic kidney disease, heart failure, and atherosclerotic disease, and baseline ED independently predicted the later development of chronic kidney disease, heart failure, atrial fibrillation, hypertension, and atherosclerotic cardiovascular disease over follow-up [81]. Notably, the relationship with incident chronic kidney disease was among the strongest, underscoring that penile vascular dysfunction reflects a systemic process spanning the heart, kidneys, and metabolic axis. Together, these findings support considering ED as a marker of heightened cardiometabolic and renal risk [78,79,80,81].

#### 11.2.2. Polycystic Ovary Syndrome

Polycystic ovary syndrome (PCOS) occupies a distinctive place among the conditions linked to metabolic syndrome, because the relationship is bidirectional rather than one of simple end-organ damage. Insulin resistance is central to both entities, making metabolic syndrome a frequent and mechanistically intertwined comorbidity of PCOS. Hyperinsulinemia stimulates androgen production by ovarian theca cells and suppresses hepatic sex hormone-binding globulin, thereby increasing circulating free androgens, while also promoting central adiposity and dyslipidemia [82]. Women with PCOS have a substantially higher prevalence of metabolic syndrome than women without PCOS, largely driven by adiposity and insulin resistance [83,84]. The long-term consequences include increased risk of T2DM, cardiovascular events, and endometrial cancer related to chronic anovulation and unopposed estrogen exposure [85,86]. A 20-year retrospective cohort documented a high age-specific burden of T2DM, myocardial infarction, and angina among women with PCOS [85].

#### 11.2.3. Colorectal Neoplasia

An association has also been reported between metabolic syndrome and colorectal precancerous polyps. This should be regarded as an associated neoplastic risk rather than a direct CKM complication. A recent meta-analysis reported a pooled HR of 1.24 (95% CI 1.01–1.51, I^2^ = 24%) [87].

#### 11.2.4. Neurocognitive and Psychiatric Associations

Cognitive deficits involving attention, processing speed, memory, and executive function have been reported in patients with MASLD, including non-cirrhotic stages, although findings are not uniform across studies [88]. Depression and anxiety are also important associated conditions. A systematic review and meta-analysis of studies using the legacy NAFLD definition documented a substantial burden of both, with considerable between-study heterogeneity [89]. Proposed mechanisms linking hepatic and brain dysfunction include insulin resistance, systemic inflammation, vascular injury, and altered gut–liver–brain signaling; impaired ammonia handling may also contribute before cirrhosis develops [88]. However, these manifestations should not automatically be equated with hepatic encephalopathy. Most clinical evidence is observational, and shared metabolic comorbidities complicate causal interpretation. The relative contribution of liver disease itself therefore remains uncertain.

### 11.3. Metabolic Risk States and Progression

#### 11.3.1. Prediabetes

Prediabetes is an intermediate state between normoglycemia and diabetes, characterized by glucose values that do not meet the diagnostic thresholds for diabetes. The oral glucose tolerance test may be more informative than glycated hemoglobin (HbA1c) for predicting future diabetes [90]. A one-hour plasma glucose concentration ≥ 155 mg/dL may identify individuals with apparently normal glucose tolerance who are at increased risk of future T2DM and adverse cardiovascular outcomes [91]. Six metabolic subphenotypes among individuals at elevated risk for T2DM have been proposed, some of which carry high risks or adverse outcomes beyond diabetes itself; a phenotype characterized by high visceral fat was associated with increased nephropathy risk [92]. These subphenotypes were derived using measures of glucose tolerance, insulin sensitivity and secretion, HDL cholesterol, liver fat, visceral and subcutaneous fat distribution, and genetic risk [92].

#### 11.3.2. Progression to T2DM

T2DM represents progression along the dysglycemic continuum and frequently coexists with other components of CKM syndrome. T2DM increases the risk of microvascular complications, including retinopathy, nephropathy, and neuropathy, as well as macrovascular complications, such as heart disease and stroke [93].

Metabolic syndrome is associated with a relative risk of incident diabetes of 3.5–5.2, depending on the definition used. Its added predictive value over the individual components—particularly fasting glucose—nevertheless remains debated [94].

A recent cohort study of 8170 adults with metabolic syndrome classified disease severity as mild, moderate, or severe according to the presence of three, four, or five diagnostic criteria, respectively. After eight years of follow-up, the incidence of T2DM increased from 21% in the mild group to 49% in the severe group [95]. Independent predictors of incident diabetes included elevated fasting plasma glucose (HR 2.13; 95% CI 1.69–2.69, *p* < 0.001), higher BMI (HR 1.33; 95% CI 1.07–1.66, *p* = 0.010) and lower HDL cholesterol (HR 1.26; 95% CI 1.01–1.59; *p* = 0.045), whereas triglycerides, blood pressure, age and sex were not independently associated with incident T2DM [95].

## 12. Therapy

### 12.1. Diet

Lifestyle modification remains the foundation of management at every stage. The Mediterranean diet—rich in vegetables, fruits, whole grains, legumes, nuts, and olive oil, with moderate fish and poultry and limited red and processed meat—is among the best-supported dietary patterns for MASLD and cardiometabolic risk reduction [12,96]. A 2025 two-year intervention centered on Mediterranean diet adherence and physical activity demonstrated improved hepatic health in patients with MASLD, including reduced intrahepatic fat [97]. In metabolic syndrome more broadly, adherence to a Mediterranean dietary pattern is associated with improvements in lipid profiles, blood pressure, insulin sensitivity, central adiposity, and inflammatory/metabolic risk markers [96].

Weight loss of clinically meaningful magnitude is a central therapeutic goal in patients with MASLD and overweight or obesity; caloric restriction, improved diet quality, and reduced ultra-processed, energy-dense foods are emphasized [12]. An emerging refinement is nutrigenetics, consistent with the gene–environment interactions described in Section 5 [28,33]. A 2025 randomized trial comparing moderately hypocaloric Mediterranean and low-fat diets in MASLD found similar improvements in hepatic steatosis and liver stiffness, with no significant modification of treatment response by *PNPLA3* genotype [98]. In patients with MASLD and obesity who otherwise meet standard indications, bariatric surgery is a recognized option [12].

### 12.2. Exercise

Structured physical activity is an independent and synergistic therapy. Randomized evidence from 2025 shows that combining Mediterranean diet adherence with structured exercise produces consistent improvements in body composition and cardiometabolic indicators across sexes in physically inactive adults, and the multicomponent design is highlighted as both effective and feasible for prevention [99]. Regular physical activity improves aerobic capacity, reduces intrahepatic fat, enhances insulin sensitivity, and lowers blood pressure, and structured exercise is being formally tested as an adjunct in MASLD patients already following a Mediterranean diet [12,100]. Dietary modification and structured exercise are complementary components of management. However, improvements in body composition and metabolic risk markers should not be equated with demonstrated reductions in long-term hepatic or cardiovascular events [12,97,99]. Resistance exercise training (RET) has emerged as an effective and largely independent modality for improving metabolic syndrome [101]. A 2025 meta-analysis of 50 randomized controlled trials enrolling 2271 participants with T2DM found that isolated RET produced significant reductions in fasting blood glucose (mean difference [MD] −7.09 mg/dL), triglycerides (MD −14.05 mg/dL), systolic (MD −4.13 mmHg) and diastolic (MD −2.03 mmHg) blood pressure, and waist circumference (MD −2.18 cm), together with a rise in high-density lipoprotein cholesterol (MD +1.86 mg/dL) [102]. Controlled trial data extend these effects to older women, in whom 12 weeks of resistance training normalized metabolic syndrome prevalence (18% at baseline to 0%) and reduced fasting glucose (−20.4%), systolic pressure (−6.2%), and C-reactive protein (−28.6%) without dietary modification [103]. Importantly, in older adults, obesity and a higher metabolic syndrome risk index were the strongest predictors of an attenuated lean mass response to a combined resistance exercise, physical activity, and nutritional intervention (r = −0.36 to −0.68). These findings are consistent with anabolic resistance in metabolically unhealthy older adults [104].

### 12.3. Medication

Pharmacological management has evolved substantially, although the strength of evidence differs across organ systems. The following sections distinguish regulatory approvals and randomized trial evidence from observational findings, guideline recommendations, and investigational approaches.

#### 12.3.1. Hepatic-Targeted Therapy

Resmetirom (Rezdiffra), an orally administered liver-selective thyroid hormone receptor-β agonist, received U.S. Food and Drug Administration (FDA) accelerated approval in March 2024—the first ever approved therapy for non-cirrhotic MASH with stage F2–F3 fibrosis [25,105]. It accelerates hepatic β-oxidation and lowers atherogenic lipoproteins while largely avoiding systemic thyrotoxic effects, and the American Association for the Study of Liver Diseases (AASLD) updated its guidance in October 2024 to incorporate it; it was subsequently granted conditional marketing authorization in the European Union in August 2025 [25,106]. Although resmetirom has demonstrated histological improvements in MASH and fibrosis, its long-term effects on liver-related clinical outcomes and cardiovascular events remain to be established [25].

#### 12.3.2. Incretin-Based Therapy

Glucagon-like peptide-1 (GLP-1) receptor agonists enhance glucose-dependent insulin secretion, regulate satiety and appetite through hypothalamic pathways, and delay gastric emptying. Tirzepatide, a dual glucose-dependent insulinotropic polypeptide (GIP)/GLP-1 receptor agonist, markedly reduced progression to T2DM in individuals with prediabetes and obesity or overweight after 176 weeks (1.3% vs. 13.3%; HR 0.07; 95% CI 0.0–0.1; *p* < 0.001), alongside substantial weight loss [107]. In the Semaglutide Effects on Cardiovascular Outcomes in People with Overweight or Obesity (SELECT) trial, semaglutide reduced the composite endpoint of cardiovascular death, non-fatal myocardial infarction, or non-fatal stroke by 20% [108].

Semaglutide, a GLP-1 receptor agonist, received FDA approval in August 2025 for MASH with moderate-to-advanced fibrosis, based on phase 3 evidence showing histological benefit [109,110]. Its cardiovascular benefits have been demonstrated in dedicated outcome trials conducted in other clinical populations and should not be interpreted as evidence of a direct cardiovascular effect mediated by improvement in MASH [108,111]. Tirzepatide, a dual GIP/GLP-1 agonist, demonstrated improvements in MASH resolution in a phase 2 randomized trial; however, long-term data on hepatic clinical outcomes and fibrosis progression remain necessary [112]. Because MASLD is highly prevalent among patients with T2DM and obesity, incretin-based therapies may provide complementary metabolic and hepatic benefits when prescribed according to their approved indications [109,112].

Beyond dual receptor agonism, retatrutide is an investigational triple GIP/GLP-1/glucagon receptor agonist with promising effects on hepatic steatosis. In a randomized, placebo-controlled phase 2a MASLD substudy involving 98 participants with obesity or overweight and without T2DM, retatrutide reduced liver fat measured by magnetic resonance imaging proton density fat fraction (MRI-PDFF). At 24 weeks, mean relative reductions were 81.4% and 82.4% with the 8 mg and 12 mg doses, respectively, compared with a 0.3% increase with placebo. However, the study did not include liver histology and therefore did not establish histological MASH resolution or fibrosis regression. These findings support further investigation, while long-term hepatic clinical benefits remain to be established [113].

#### 12.3.3. Cardio-Renal Protective Therapy

Observational studies suggest possible hepatic benefits of sodium–glucose co-transporter 2 (SGLT2) inhibitors in patients with MASLD. A propensity score-matched retrospective analysis reported associations between SGLT2 inhibitor exposure and lower mortality and incidence of advanced liver disease [114]. Another retrospective study found that combined GLP-1 receptor agonist and SGLT2 inhibitor therapy was associated with lower mortality than SGLT2 inhibitor monotherapy [115]. These findings remain susceptible to residual confounding, selection bias, and differences in treatment allocation and follow-up. They therefore do not establish causal hepatic or survival benefits or prove the superiority of combination therapy.

In contrast to these observational findings, a meta-analysis of randomized dapagliflozin trials across kidney, cardiovascular, and metabolic conditions found reductions in heart failure hospitalization (HR 0.72; 95% CI 0.66–0.79; *p* < 0.001), cardiovascular mortality (HR 0.89; 95% CI 0.80–0.98; *p* = 0.015) and all-cause mortality (HR 0.88; 95% CI 0.80–0.97; *p* = 0.008) [116].

Guideline recommendations support SGLT2 inhibitor use in eligible patients with heart failure, CKD, or T2DM, according to the specific indication and clinical context [1]. These recommendations should be distinguished from the observational hepatic findings discussed above. Randomized outcome trials have demonstrated cardiovascular benefit with selected GLP-1 receptor agonists and kidney protection with semaglutide in patients with T2DM and CKD in the Evaluate Renal Function with Semaglutide Once Weekly (FLOW) [111,117]. Finerenone demonstrated kidney and cardiovascular benefits in patients with T2DM and CKD in Finerenone in Reducing Kidney Failure and Disease Progression in Diabetic Kidney Disease (FIDELIO-DKD) and Finerenone in Reducing Cardiovascular Mortality and Morbidity in Diabetic Kidney Disease (FIGARO-DKD). In Finerenone Trial to Investigate Efficacy and Safety Superior to Placebo in Patients with Heart Failure (FINEARTS-HF), it reduced the composite of total worsening heart failure events and cardiovascular death in patients with mildly reduced or preserved ejection fraction [111]. These findings support treatment within the respective clinical indications, not uniform efficacy across all CKM stages or established hepatic protection.

Non-invasive neuromodulation targeting peripheral neural pathways is being investigated for effects on appetite, inflammation, and metabolic regulation. The supporting literature is largely mechanistic or preclinical, with limited clinical evidence and substantial heterogeneity between techniques. Its efficacy and long-term safety in CKM syndrome and MASLD remain insufficiently established to support routine clinical use [118,119,120].

Bariatric surgery can induce substantial and durable improvement in metabolic syndrome. Its effects extend beyond weight loss and include changes in incretin and appetite-regulating hormones, such as increased GLP-1 and peptide YY, reduced leptin concentrations, increased adiponectin, improved insulin sensitivity, and favorable shifts in the gut microbiota [121].

## 13. Public Health Burden, Limitations and Future Directions

Because CKM syndrome and MASLD are both highly prevalent and contribute to heart failure, kidney disease, and end-stage liver disease, their combined economic and public health burden is immense and rising [1,19,20]. A 2026 GBD analysis projects that approximately 1.8 billion people may be living with MASLD by 2050 [21]. An integrated clinical approach linking CKM assessment with evaluation for MASLD may improve multidisciplinary screening and prevention by treating underutilized albuminuria testing and non-invasive liver fibrosis assessment as a shared responsibility of primary care, cardiology, endocrinology, nephrology, and hepatology [1].

Some epidemiological estimates continue to rely on legacy NAFLD data interpreted within the newer MASLD framework [14]. In lower-resource settings, limited healthcare access may complicate disease detection and burden estimation [21]. The genetic dissociation between hepatic fat alleles and cardiovascular risk complicates causal inference and risk communication [34,35]. miRNA biomarkers, although promising for early non-invasive diagnosis across all three organ systems, remain heterogeneous across studies and are not yet validated for routine clinical use in large prospective cohorts [62,63,64]. Finally, real-world finerenone use remains modest relative to the supporting trial evidence; U.S. prescribing analyses from 2021 to 2024 show increasing but still specialty-concentrated uptake [122].

Priorities for the coming years follow directly: evaluation of whether and how hepatic biomarkers should be incorporated into future CKM assessment [1]; validation and standardization of circulating and urinary miRNA panels [62,63,64]; phenotype- and genotype-guided (precision) selection of diet and pharmacotherapy [27,34]; equitable expansion of low-cost non-invasive diagnostics to under-resourced regions [21]; and pragmatic strategies to translate guideline-directed, multi-organ combination therapy into front-line practice [1,122].

## 14. Conclusions

Recent literature supports close biological and clinical interactions among cardiovascular, renal, metabolic, and hepatic diseases through shared mechanisms, including dysfunctional adiposity, insulin resistance, chronic inflammation, and fibrosis. Although these interactions support integrated clinical assessment, CKM remains the established guideline-based framework, whereas the incorporation of hepatic disease represents an evolving conceptual extension that is not currently included in formal CKM staging. Genetics (*PNPLA3*, *TM6SF2*, and polygenic scores) and emerging circulating miRNA biomarkers are moving the field toward precision diagnosis, while distinct phenotypes argue against uniform management. Therapeutic advances include approved treatments for selected patients with MASH and cardiovascular and renal benefits demonstrated with SGLT2 inhibitors, selected incretin-based therapies, and finerenone in defined trial populations. These findings support indication-based, integrated care alongside dietary modification and physical activity, but should not be interpreted as uniform protection across all organ systems. The principal unmet needs include determining whether and how hepatic markers should be incorporated into future CKM staging, expanding access to non-invasive diagnostics in low-resource regions, and closing the gap between evidence and real-world prescribing.

## Figures and Tables

**Table 1:** Revised NCEP ATP III criteria for Metabolic Syndrome.

Criterion	Threshold
Waist circumference	≥102 cm (men)/≥88 cm (women)
Triglycerides	≥150 mg/dL (or on treatment)
HDL cholesterol	<40 mg/dL (men)/<50 mg/dL (women)
Blood pressure	≥130/85 mmHg (or on treatment)
Fasting glucose	≥100 mg/dL or treatment for elevated glucose

HDL, high-density lipoprotein, NCEP ATP III, National Cholesterol Education Program Adult Treatment Panel III.

**Table 2:** Historic definitions of metabolic syndrome.

Definition	Mandatory Component	Diagnostic Rule	References
NCEP ATP III (2001/revised 2005)	None	Any 3 of 5 criteria	[3,4]
IDF (2005)	Central obesity	Central obesity plus any 2 of the remaining 4 criteria	[5,6]
WHO (1998/1999)	Insulin resistance	Insulin resistance plus any 2 additional criteria	[7,8]
EGIR (1999)	Hyperinsulinemia	Hyperinsulinemia plus any 2 additional criteria	[9]
Harmonized definition (2009)	None	Any 3 of 5 criteria *	[10]

* Waist-circumference thresholds are population- and country-specific. EGIR, European Group for the Study of Insulin Resistance; IDF, International Diabetes Federation; NCEP ATP III, National Cholesterol Education Program Adult Treatment Panel III; WHO, World Health Organization.

**Table 3:** Comparison of NAFLD, MAFLD, and MASLD definitions.

Term	Definition	Key Difference	References
NAFLD (legacy nomenclature)	Hepatic steatosis with exclusion of significant alcohol consumption (<20 g/day in women, <30 g/day in men) and other recognized causes	Diagnosis of exclusion; “non-” and “fatty” considered imprecise and stigmatizing	[11,13]
MAFLD (proposed in 2020)	Hepatic steatosis plus overweight/obesity, T2DM, or at least two metabolic-dysregulation criteria	Positive criteria; alcohol not exclusionary; allows dual etiology	[13]
MASLD (introduced in 2023)	Hepatic steatosis plus at least one of five cardiometabolic criteria, with alcohol consumption below the MetALD range (<20 g/day in women/<30 g/day in men)	Requires at least one cardiometabolic criterion and forms part of broader SLD nomenclature; introduces MetALD for intermediate alcohol intake (20–50 g/day in women and 30–60 g/day in men)	[11,12]
Practical overlap	High overlap between NAFLD and MASLD in most cohorts	Unlike NAFLD, MASLD explicitly requires at least one cardiometabolic risk factor	[12,14]

MAFLD, metabolic dysfunction-associated fatty liver disease; MASLD, metabolic dysfunction-associated steatotic liver disease; MetALD, metabolic dysfunction- and alcohol-related steatotic liver disease; NAFLD, non-alcoholic fatty liver disease; SLD, steatotic liver disease; T2DM, type 2 diabetes mellitus.

**Table 4:** Non-invasive fibrosis risk stratification.

Test	Low Risk (Rule-Out)	Indeterminate	High Risk (Refer to Hepatology)	References
FIB-4 (age <65 years)	<1.30	1.30–2.67	>2.67	[54]
FIB-4 (age ≥65 years)	<2.00	2.00–2.67	>2.67	[54,56]
NAFLD Fibrosis Score (NFS)	<−1.455 (<0.12 if ≥65 years)	−1.455 to 0.676 (0.12 to 0.676 if ≥65 years)	>0.676	[56,57]
ELF score	<7.7	7.7 to <9.8	≥9.8	[54,58]
VCTE/FibroScan LSM	<8 kPa	8 to <12 kPa	≥12 kPa	[54,59]

ELF, Enhanced Liver Fibrosis; FIB-4, Fibrosis-4; LSM, Liver stiffness measurement; NAFLD, non-alcoholic fatty liver disease; NFS, NAFLD Fibrosis Score; VCTE, Vibration-controlled transient elastography.

## Data Availability

No new data were created or analyzed in this study. Data sharing is not applicable to this article.

## Abbreviations

The following abbreviations are used in this manuscript:

AASLD	American Association for the Study of Liver Diseases
ACC	American College of Cardiology
ADA	American Diabetes Association
AGE	Advanced glycation end-product
AHA	American Heart Association
ASN	American Society of Nephrology
BMI	Body mass index
CI	Confidence Interval
CKD	Chronic kidney disease
CKM	Cardiovascular-kidney-metabolic
CVD	Cardiovascular disease
DALY	Disability-adjusted life-year
ED	Erectile dysfunction
EGIR	European Group for the Study of Insulin Resistance
eGFR	Estimated glomerular filtration rate
ELF	Enhanced Liver Fibrosis
FDA	Food and Drug Administration
FIB-4	Fibrosis-4
FIDELIO-DKD	Finerenone in Reducing Kidney Failure and Disease Progression in Diabetic Kidney Disease
FIGARO-DKD	Finerenone in Reducing Cardiovascular Mortality and Morbidity in Diabetic Kidney Disease
FINEARTS-HF	Finerenone Trial to Investigate Efficacy and Safety Superior to Placebo in Patients with Heart Failure
FLOW	Evaluate Renal Function with Semaglutide Once Weekly
GBD	Global burden of disease
GIP	Glucose-dependent insulinotropic polypeptide
GLP-1	Glucagon-like peptide 1
GLUT4	Glucose transporter type 4
HbA1c	Glycated hemoglobin
HCC	Hepatocellular carcinoma
HDL	High-density lipoprotein
HFpEF	Heart failure with preserved ejection fraction
HR	Hazard ratio
IDF	International Diabetes Federation
IL-6	Interleukin-6
IL-1β	Interleukin-1β
IL-1Ra	Interleukin-1 receptor antagonist
KNHANES	Korea National Health and Nutrition Examination Survey
LDL	Low-density lipoprotein
LSM	Liver stiffness measurement
MAFLD	Metabolic dysfunction-associated fatty liver disease
MASLD	Metabolic dysfunction-associated steatotic liver disease
MASH	Metabolic dysfunction-associated steatohepatitis
MD	Mean difference
MetALD	Metabolic dysfunction- and alcohol-related steatotic liver disease
miRNA	Micro RNA
MRI-PDFF	Magnetic resonance imaging-proton density fat fraction
MUHNW	Metabolically unhealthy normal-weight
MUHO	Metabolically unhealthy obese
NAFLD	Non-alcoholic fatty liver disease
NASH	Non-alcoholic steatohepatitis
NCEP ATP	National Cholesterol Education Program Adult Treatment Panel
NHANES	National Health and Nutrition Examination Survey
NFS	NAFLD Fibrosis Score
OR	Odds ratio
PCOS	Polycystic ovary syndrome
PROSPER	Prospective Study of Pravastatin in the Elderly at Risk
RAAS	Renin–angiotensin–aldosterone system
RET	Resistance exercise training
ROS	Reactive oxygen species
RR	Relative risk
SELECT	Semaglutide Effects on Cardiovascular Outcomes in People with Overweight or Obesity
SGLT2	Sodium-glucose co-transporter 2
SLD	Steatotic liver disease
T2DM	Type 2 Diabetes mellitus
TNF-α	Tumor necrosis factor-α
TyG	Triglyceride-glucose
VEGF	Vascular endothelial growth factor
VCTE	Vibration-controlled transient elastography
WHO	World Health Organization
